# Spheroid Culture Differentially Affects Cancer Cell Sensitivity to Drugs in Melanoma and RCC Models

**DOI:** 10.3390/ijms23031166

**Published:** 2022-01-21

**Authors:** Aleksandra Filipiak-Duliban, Klaudia Brodaczewska, Arkadiusz Kajdasz, Claudine Kieda

**Affiliations:** 1 Laboratory of Molecular Oncology and Innovative Therapies, Military Institute of Medicine, 04-141 Warsaw, Poland; kbrodaczewska@wim.mil.pl; 2 Postgraduate School of Molecular Medicine, Medical University of Warsaw, 02-091 Warsaw, Poland; 3 Department of RNA Metabolism, Institute of Bioorganic Chemistry, Polish Academy of Sciences, Noskowskiego 12/14, 61-704 Poznan, Poland; akajdasz@ibch.poznan.pl; 4 Laboratory of Human Molecular Genetics, Faculty of Biology, Institute of Molecular Biology and Biotechnology, Adam Mickiewicz University Poznan, 61-614 Poznan, Poland; 5 Center for Molecular Biophysics UPR 4301 CNRS, CEDEX 2, 45071 Orleans, France; ckieda@wim.mil.pl

**Keywords:** RCC, glutathione-s-transferases, 3D, spheroids, melanoma, drug-resistance, doxorubicin, cisplatin, everolimus, cytochrome

## Abstract

2D culture as a model for drug testing often turns to be clinically futile. Therefore, 3D cultures (3Ds) show potential to better model responses to drugs observed in vivo. In preliminary studies, using melanoma (B16F10) and renal (RenCa) cancer, we confirmed that 3Ds better mimics the tumor microenvironment. Here, we evaluated how the proposed 3D mode of culture affects tumor cell susceptibility to anti-cancer drugs, which have distinct mechanisms of action (everolimus, doxorubicin, cisplatin). Melanoma spheroids showed higher resistance to all used drugs, as compared to 2D. In an RCC model, such modulation was only observed for doxorubicin treatment. As drug distribution was not affected by the 3D shape, we assessed the expression of MDR1 and mTor. Upregulation of MDR1 in RCC spheroids was observed, in contrast to melanoma. In both models, mTor expression was not affected by the 3D cultures. By NGS, 10 genes related with metabolism of xenobiotics by cytochrome p450 were deregulated in renal cancer spheroids; 9 of them were later confirmed in the melanoma model. The differences between 3D models and classical 2D cultures point to the potential to uncover new non-canonical mechanisms to explain drug resistance set by the tumor in its microenvironment.

## 1. Introduction

One of the limiting factors to achieve cures for cancer and other diseases is drug resistance. This phenomenon is well known, universal, and its development is almost inevitable [1,2]. Some of the tumors are characterized by primary drug resistance, while others become unresponsive to treatment during chemotherapy [3]. There are multiple mechanisms underlying cancer unresponsiveness and/or resistance to treatment, reviewed elsewhere [4]. Briefly, they include changes in the drug target (receptor or pathway expression or mutation) [5], induction of metabolism or efflux of drugs [6], interruption of cell death and activation of survival mechanisms [7], and reprogramming the tumor microenvironment to promote tumor growth [8]. One of the most frequent causes of cancer cell resistance is mediated by multi drug resistance (MDR). It occurs when cancer cells gain resistance to drugs that are functionally and structurally unassociated to those to which they were previously exposed [3]. A key role played by MDR is the increased activity of drug efflux pumps. This is typically the effect of the ATP-binding cassette (ABCB1/MDR1) [3,9]. This plasma membrane glycoprotein is often overexpressed in cancers which do not respond to chemotherapy [3,10]. Other important mechanisms involve the modulation of apoptosis/autophagy, miRNA regulation, and DNA damage and repair among epigenetic regulations [3]. In the development of MDR are the non-cellular components of tumor microenvironment (TME), among which are the extracellular matrix, pH, oxygenation, soluble factors (cytokines), and vascular-endothelial growth factor (VEGF) [6]. The estimation of MDRs activity during the course of treatment as well as for the prevision of the treatment efficacy are often based on the potential a drug has to avoid the induction of resistance. A classic example is provided by daunorubicin which is often excluded of the highly aggressive cancer stem cells (CSC) [3,9,10,11]. Induction of those and other mechanisms reduces the efficacy of both chemotherapeutics and targeted therapy and inevitably leads to cancer resistance and progression.

In this line, the activity of a chemotherapeutic molecule is traditionally evaluated using the common two-dimensional cell cultures, which are far from reproducing the conditions of the original tumor microenvironment. For this reason, many drugs evaluated using such models proved to be clinically ineffective [12]. In our preliminary studies, we proposed 3D model conditions for the culture of renal (RenCa) and melanoma (B16F10) cancer cells. By comparing the 3D cultures with in vivo obtained tumors, we showed that spheroids better mimic the in vivo tumor characteristics than 2D cultures (“Spheroid culture models imitating the tumor microenvironment of renal and melanoma cancer”, submitted).

Application of 3D culture model, as a way to reconstitute in vitro some aspects of TME, is now widely discussed as a proper model for preliminary research in tumor biology [13]. As an increasing number of therapeutic strategies focus on non-cancer components of TME (such as immune cells, stromal cells, hypoxia), which shape the progression of the disease, new tools are being developed to enable modeling more accurately the responses of tumor and the host [14,15]. They include the incorporation into spheroid culture non-cellular components, such as ECM proteins [16], but also the creation of much more complicated models containing stromal cells, such as fibroblasts [17], endothelial cells [18], or even immune cells [19,20]. All these elements highly influence spheroid, hence potentially a tumor, response to treatment [21]. 3D/spheroid culture is a well developing area of oncology and various techniques are being developed [22]. Yet, this abundance of 3D culture methods and protocols causes a lot of confusion as to which to choose in a particular study, taking into consideration not only the modeled tumor, but also technical resources of a particular research group. Nonetheless, the superiority of organoid cultures over standard monolayers [23], encourages to test numerous variants of 3D cultures as tools to study molecular and cellular mechanisms that determine the progression of cancer. This might account for a higher accuracy of selection of drugs that pass into clinical trials and the more rapid development of personalized medicine [24].

The aim of the current study was to evaluate how the proposed, simple, 3D culture conditions affect the tumor cell susceptibility to anti-cancer drugs. We used several commonly applied chemotherapeutics which are differing in their mechanism of action, such as everolimus which binds to intracellular, FKBP-12 forming an inhibitory complex with mTOR complex 1 (mTORC1), thus inhibiting mTOR kinase activity and consequently works ultimately as an inhibitor of the PI3K/AKT/mTOR pathway [25]; cisplatin—platinum-based drug which affects proliferation and apoptosis by cross-linking the DNA bases [26]; doxorubicin—which increases death of cells by intercalation into the DNA and inhibition of topoisomerase II modifying the chromatin structure, as well as by generation of free radicals and oxidative damage to biomolecules [27]. We observed that despite the induction of a similar set of drug resistance-related genes in two distinct cancer models upon culture conditioning, the susceptibility to cytotoxic agents was not directly correlated, showing the engagement of new pathways that could be uncovered using the proposed 3D models.

## 2. Results

### 2.1. Spheroid Formation

**Figure 1:**
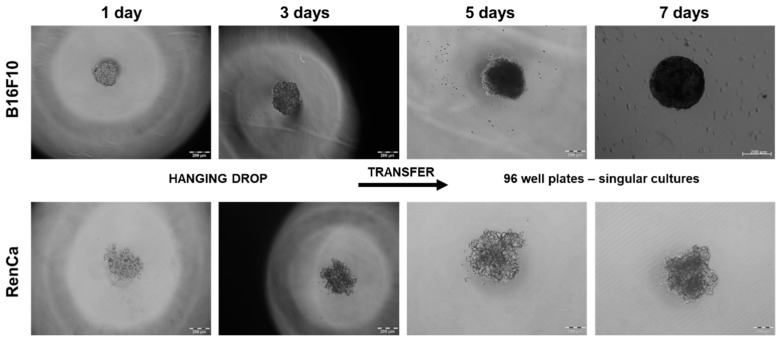
Spheroid formation for 7 days. B16F10 and RenCa cell lines were seeded 500 of cells per drop in RPMI medium supplemented with methylcellulose; 3 days after cultivation in hanging drops, cell aggregates were transferred in agarose coated 96-well plates, and cultivated for another 4 days.

As in B16F10, seeding density does not have a high influence on spheroid shape (data not shown), in the RenCa model, low or to high number of cells affected cell viability or shape, respectively (Supplementary Figure S1). In order to obtain regular spheroids with a diameter higher or equal to 400 µM, the seeding density was optimized to 500 of cells/drop and a cultivation time of up to 7 days for both cell lines. After three days of culture, irregular aggregates were observed, and regular spheroids were observed after seven days of cultures in both cell lines (Figure 1).

### 2.2. 3D Cultures Show Different Sensitivity to Chemotherapy

**Figure 2:**
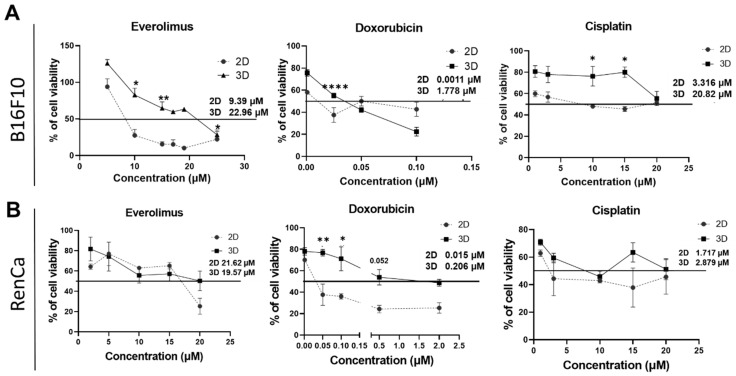
Sensitivity of cells in 2D and 3D culture. (**A**)—sensitivity to everolimus, doxorubicin, and cisplatin of B16F10 cell line in 2D and 3D culture conditions as assessed by the use of the alamarBlue method. (**B**)—sensitivity to everolimus, doxorubicin, and cisplatin of RenCa cell lines in 2D and 3D cultures assessed using alamarBlue method. Statistical analysis was performed by One-Way ANOVA/Tukey test or Kruskal–Wallis/Dunn’s test—* *p* < 0.05, ** *p* < 0.0021, **** *p* < 0.0001, *N* ≥ 3 (2D—control).

To asses if 3D cultures will affect drug sensitivity, we evaluated cell viability (using alamarBlue assay) after exposure to commonly used drugs, such as everolimus, doxorubicin, and cisplatin. In the melanoma cancer model, spheroids showed higher resistance to everolimus; in 2D cultures, the IC_50_ value was 9.39 µM and in 3D cultures, it reached 22.96 µM (Figure 2A). In the renal cancer model, no significant differences between monolayer (IC_50_ 21.62) and 3D culture (IC_50_ 19.57) were observed for these drugs (Figure 2B). These results were confirmed by staining drug treated cells with propidium iodide showing that incorporation into the DNA concerned a higher proportion of B16F10 cells when cultured in 2D than in spheroids (Supplementary Figure S2). B16F10 spheroid cultures were also less sensitive to cisplatin and doxorubicin as IC_50_ levels were higher than in 2D cultures for Cisplatin: 3D, 20.82 µM; 2D 3.316 µM, and to lesser extent for Doxorubicin: 3D, 1.778 µM; 2D, 0.0011 µM (Figure 2A). In the renal cancer model, an altered sensitivity was observed to doxorubicin only; IC_50_ levels for that model were higher for spheroids: 3D, 0.206 µM; 2D, 0.015 µM (Figure 2B).

### 2.3. Distribution of the Drug Is Not Affected by Sphere Shape

**Figure 3:**
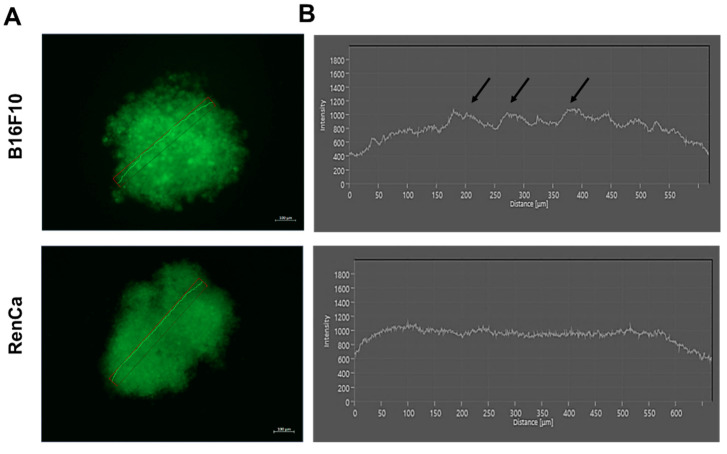
Drug distribution in the spheres. Immunofluorescent picture of doxorubicin (0.001 µM) treated melanoma and renal cancer spheres after 48 h treatment—(**A**). Histograms representing drug distribution in the cross-section of the sphere of melanoma and renal cancer model—(**B**).

As a higher resistance to all tested drugs was observed in 3D melanoma cultures than in 2D, we assessed whether these differences result from the conditions applied by its spheroidic architecture altering the drug distribution. Doxorubicin is a fluorescent molecule (0.001 µM), its distribution was thus estimated (Figure 3A,B), by assessing fluorescence intensity at different points in the spheres after incubation of the spheres with drug for 48 h. In the renal model, no significant change was observed as the fluorescent signal was homogenous throughout the sphere. In the melanoma model, several peaks were distinguishable along the fluorescence signal histogram, indicating drug accumulation. The drug penetrated the whole mass of the 3D structure, including its core (Figure 3A,B).

### 2.4. 3D Culture Conditions Can Affect MDR1 Expression Differently in RCC and Melanoma, but Have No Effect on mTor Expression

**Figure 4:**
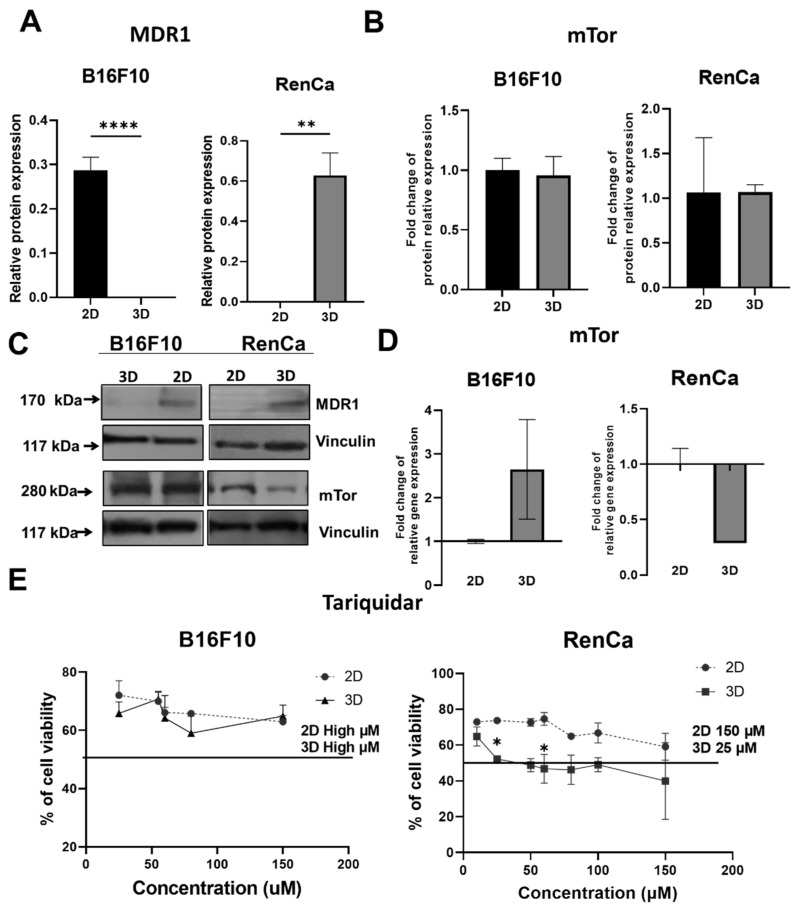
Regulation of MDR1 and mTor expression. (**A**)—Relative to 2D protein expression of MDR1 of melanoma and renal cancer cells, Vinculin served as loading control. (**B**)—Relative to 2D protein expression of mTor of melanoma and renal cancer cells, Vinculin served as loading control. (**C**)—Detection of MDR1 and mTor by western blot. (**D**)—The gene expression of *mtor* was determined by quantitative RT-PCR (qRT-PCR); β-actin served as an internal control. (**E**)—Sensitivity to tariquidar in various concentrations estimated by Alamar blue assay—(**D**). Statistical analyses were performed by One-Way NOVA/Tukey test or Kruskal–Wallis/ Dunn’s test—* *p* < 0.05, ** *p* < 0.0021, **** p < 0.0001, *N* ≥ 3 (2D—control).

Drug resistance is often due to MDR1, a glycoprotein which is widely expressed in cancer cells, ensuring exportation of the drugs out of the cells [28]. We assessed if differences in the sensitivity to drugs might be related to MDR1 expression. In the B16F10 model, the MDR1 protein bands were visible only for 2D cultures, while no expression of MDR1 was detected in 3D culture lysates. Oppositely, in the RenCa model, no MDR1 protein was detected in 2D, but it was induced by spheroid culture (Figure 4A,C). To assess for the role of MDR1 for cell viability, we assessed the cell sensitivity to Tariquidar which is an MDR1 inhibitor [29]. Viability of B16F10 cells in both culture conditions was not affected; both in MDR1-expressing 2D and MDR1-negative 3D cells (Figure 4E). In the case of the renal cancer cells model, the 3D cultures were more sensitive to Tariquidar than 2D cultures (Figure 4E)—the IC_50_ levels for 3D was 25 µM, while for 2D, the IC_50_ was 150 µM. These results were confirmed by staining drug treated cells with propidium iodide. More dead RenCa cells were observed after exposure to Tariquidar in 3D culture models (Supplementary Figure S2). The mammalian target of rapamycin (mTor) is an important regulator of proliferation, differentiation, apoptosis, and tumorigenesis [30]. mTor is very strongly associated with tumor malignancy in melanoma and renal cancer [31,32]. Consequently, we assessed if the modulation of mTor protein and its gene expression in 2D and 3D cultures for both cancer models might explain the differences in the response to everolimus. In the melanoma model, no significant change for both protein and gene expression was observed between the culture conditions (Figure 4B–D). In the renal cancer model, a downregulation of gene expression was observed in 3D cultures, but this effect was not maintained at the level of protein (Figure 4B–D).

### 2.5. 3D Cultures Modulate Drug Resistance Genes

**Figure 5:**
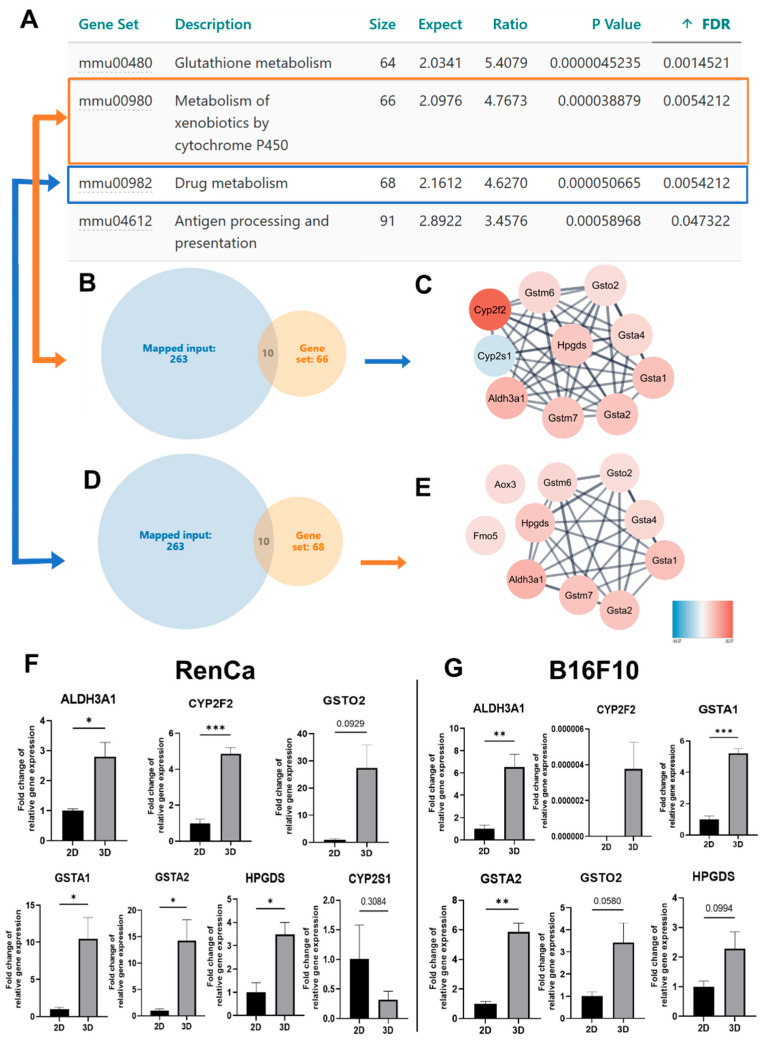
Modulation of drug related genes by 3D cultures. (**A**)—Enrichment analysis based on Gene Ontology pathway shows the top 4 activated processes in both cell types (web-based gene set analysis toolkit enrichment method: ORA; organism: mus musculus). (**B**)—screening of genes related with metabolism of xenobiotics. (**D**)—screening of genes related with metabolism of drugs. (**C**,**E**)—Functional enrichment protein network performed in Cytoscape software (v.3.8.0). (**F**)—The gene expression of *Cyp2s1*, *Cyp2f2*, *Gstm6*, *Gsto2*, *Gsta4*, *Hpgds*, *Gsta1*, *Gsta2*, *Gstm7*, *Aldh31a* for RenCa cells was determined by quantitative RT-PCR (qRT-PCR); β-actin served as a quantitative internal control. (**G**)—The gene expression of *Cyp2s1*, *Cyp2f2*, *Gstm6*, *Gsto2*, *Gsta4*, *Hpgds*, *Gsta1*, *Gsta2*, *Gstm7*, *Aldh31a* for B16F10 cells was determined by quantitative RT-PCR (qRT-PCR); β-actin served as a quantitative internal control. Statistical analysis was performed by One-Way ANOVA/Tukey test or Kruskal–Wallis/ Dunn’s test—* *p* < 0.05, ** *p* < 0.0021, *** *p* < 0.0002, *N* ≥ 3 (2D—control).

Next-generation sequencing (NGS) of 2D and 3D cultures from renal cancer model was performed to screen for other potential drug resistance modulators (Figure 5). Among all 263 (data not shown) deregulated genes in the 3D model, 10 genes related with metabolism of xenobiotics by cytochrome p450 were changed: downregulated—*Cyp2s1* and upregulated—*Cyp2f2*, *Gstm6*, *Gsto2*, *Gsta4*, *Hpgds*, *Gsta1*, *Gsta2*, *Gstm7*, *Aldh31a*. 10 related with drug metabolism were also found (Figure 5): upregulated—*Aox3*, *Fmo5*, the remaining genes modulated in this pathway were included in the above “metabolism of xenobiotics by cytochrome p450 pathway” (Figure 5B–E). We validated those results and assessed if similar responses will be observed in melanoma model using qRT-PCR method (Figure 5). In both models, similar tendencies as observed in NGS data, were for: *Cyp2f2*, *Gstm6*, *Gsto2*, *Gsta4*, *Hpgds*, *Gsta1*, *Gsta2*, *Gstm7*, *Aldh31a* genes (Figure 5). In contrast to renal cancer model, no expression of *Cyp2s1* could be detected in the melanoma cells, in both culture conditions (Figure 5).

## 3. Discussion

In separate studies, we showed that our proposed 3D cultures better mimic the in vivo tumor characteristic (“Spheroid culture models imitating the tumor microenvironment of renal and melanoma cancer”, submitted). This suggested that the model could be used as a tool in pre-clinical studies instead of mouse models for drug screening. Here, we assessed how 3D cultures will respond to chemotherapy treatment compared to standard monolayer cultures, as higher drug resistance to drugs were already observed for spheroid cultures in other cancer models [11].

Using drugs commonly used in cancer treatment such as everolimus, cisplatin, and doxorubicin [32,33,34], we assessed various cell responses by calculating IC_50_ values for 3D and 2D cultures. IC_50_ values obtained in vitro are different depending on a cell line (e.g., Cisplatin IC_50_ for human cell lines can range from 3.3 to 58 uM [35] and similar values were calculated in our study) and are often clinically relevant (eg. Cisplatin blood concentrations vary from 0.6–12 uM) [36,37]. Comparison of IC_50_ values showed that 3D cultures from melanoma model show higher resistance to some of used drugs (Figure 2A). 3D increased the IC_50_ value for everolimus 2.5 times, while for cisplatin, the change was over 6 fold, reaching 20 uM, which is similar to induced resistance by long term in vitro exposure to cisplatin in other models [38]. For doxorubicin, we did not observed such a strong effect (Figure 2A). However, in the renal cancer 3D model, tendencies were opposite; higher resistance only for doxorubicin was observed (Figure 2B). In monolayers, the cells seemed to be very sensitive to this drug with low IC_50_ compared to other cell lines [39]. Nonetheless, we used doxorubicin only as molecule to model drug responses, as it is not an established treatment in RCC [40]; therefore, we do not conclude about its therapeutic potential. However, we could evidence that 3D culture evidently modulated cell response to doxorubicin. For everolimus and cisplatin, no significant differences were shown in the RCC model. Studies have shown that spheroid three-dimensional shape may affect drug penetration and these phenomena may explain the higher resistance to chemotherapy treatment [41]. However, after staining the cells using doxorubicin at low, poorly toxic concentration, we measured the drug distribution and in both models, it was quite even. In the 3D melanoma model, several peaks were observed which may actually suggest accumulation of the drug, although it did not affect cell susceptibility to this drug. These results suggest that higher resistance to doxorubicin in the RCC model results from activation of particular cellular mechanisms rather than limited drug distribution and cell exposure. This might be the case also for altered sensitivity to cisplatin and everolimus in melanoma, although, due to differences in chemical structure, doxorubicin cannot equally model penetration of those drugs with certainty. Therefore, we screened for typical drug resistance mechanisms that could be variably induced in the two models.

ABC transporters such as P-glycoprotein (P-gp/MDR1/ABCB1) have an important role in the drugs’ extrusion from the cells [29]. Over expression is often observed in cancer cells, and this phenomenon can be a cause of failure of anticancer drug therapy [42]. Expression of MDR1 is often upregulated in 3D cultures in cancer cells [42,43,44]. We observed such a phenomenon in renal cancer model in contrast to melanoma, where MDR1 was expressed only in 2D cultures and was not detected in cells from 3Ds (Figure 4A,C). Upregulation of MDR1 in renal cancer spheroids suggests that they should be more resistant to chemotherapy; however, we observed higher resistance to doxorubicin only (Figure 2B). Absence of MDR1 in melanoma 3Ds might partly explain the drug accumulation observed microscopically after doxorubicin treatment (Figure 3A,B). We assessed using tariquidar if the inhibition of MDR1 affects cell viability (Figure 4E). Spheroids from renal cancer, in which upregulation of MDR1 was observed, were more sensitive to tariquidar, while 2D cultures seemed not to be affected (Figure 4E). These results suggest that, in the 3D structures, MDR1 protein is important for cell survival, although it does not universally predict resistance to chemotherapeutics, as cisplatin sensitivity was not altered and it can be reduced by MDR activation in other models [45,46]. In our preliminary studies, we showed that 3D cultures of RenCa cells show a larger population of Cancer stem cells CSC (“Spheroid culture models imitating the tumor microenvironment of renal and melanoma cancer”, in review). As CSCs are an important mediators of cancer resistance [47], a tool allowing to model their activity in vitro is of big importance CSCs were characterized by upregulation of ABCB transporters such as MDR1 [11,48] and the presence of cancer stem cells correlates with ability of forming spheroids [49,50]. Despite the presence of MDR1 protein in 2D cultures in melanoma, cancer cells in both culture conditions were not affected by tariquidar treatment (Figure 4C).

To verify if everolimus sensitivity is modulated by the alteration the drug target expression in 3D culture, we examined mTOR levels in monolayer and spheroid cultured cells. The mammalian target of rapamycin (mTOR) plays important roles in controlling cell behavior, as it takes part in cell proliferation, survival, invasion, and angiogenesis [32,51]. mTor is often mutated in cancer cells, among which melanoma and renal cancer [32,51]. Everolimus is commonly used drug which targets mTor [32]. As increased resistance of B16F10 3D to everolimus could not be explained by induction of MDR1 nor potentially altered drug distribution, we assessed the level of drug target, mTor, in both culture conditions. In both models, the protein expression of mTor was not affected by 3D culture. In renal cancer, we observed the down regulation of *mTor* gene expression. Therefore, the level of mTor could not predict the susceptibility to everolimus. However, it was not proved that mTOR level correlates with cell sensitivity to its inhibitors; therefore, experimental confirmation of cancer cell response to drugs might be needed to predict patient outcome in case of mTor inhibitors.

As none of the standard drug resistance mechanisms could explain distinct susceptibility of 3D models, NGS was performed to screen for other potential drug resistance modulators (Figure 5). In renal cancer spheroids, 10 genes related with metabolism of xenobiotics by cytochrome p450 were identified, and similar regulation of 9 of those genes were later confirmed in melanoma model (Figure 5). Cytochromes P450 (CYPs) and the glutathione-S transferases (GST) are important regulators of drug resistance. CYPs as well as GST known as phase I and phase II detoxification enzymes, respectively, and are capable of catalyzing the oxidative biotransformation of most drugs and other lipophilic xenobiotics [52,53,54]. Glutathione-S transferases can cooperate with ABCB transporters such as MDR1 to confer resistance to the cyto- and genotoxicities of some anticancer drugs and carcinogens [55]. In the renal cancer model, we observed an upregulation of MDR1 in 3D cultures, but they were equally sensitive to the doxorubicin treatment in monolayer. The mechanisms of xenobiotic detoxification are crucial elements for cell exclusion processes of several drugs, carcinogens, and other toxins [52,56]. It was shown that antioxidants such as Piper betel leaves inhibit GST isoforms, which in turn enhances the sensitivity of cancer cells to cisplatin [57]. These findings suggest that targeting cytochrome p450 component’s during cancer therapy may increase drug efficacy. However, as cytochrome p450 pathway was equally induced in both models, we could not explain which mechanism mediates the differential resistance of 3D models to doxorubicin or cisplatin and everolimus.

Other authors [58] reported that seemingly similar 3D spheroids show differential susceptibility to drugs that cannot be explained by molecular or metabolically variables. Rather, particular features of cells (or later, patients) determine treatment outcome. This points to the fact that when no molecular predictive factors for chemotherapeutics choice are available, experimental drug screening on patient-derived cells should be used as a personalized medicine approach [59]. Our model constitutes an accessible, economical tool that could be used for such purpose. It does not require sophisticated techniques nor equipment, making it feasible in many, non-specialized healthcare institutions.

## 4. Materials and Methods

### 4.1. Cell Lines

The tests were carried out with murine melanoma B16-F10 (given by Prof. J. Dulak, Department of Medical Biotechnology, Faculty of Biochemistry, Biophysics and Biotechnology, Jagiellonian University, Cracow, Poland; authenticated in 2021, ATCC) and renal carcinoma RenCa (CRL-2947™, ATCC, Manassas, VA, USA) cell lines. Cells were maintained in standard culture conditions: humidified atmosphere containing 5% CO_2_ at 37 °C, using RPMI medium—RPMI-1640 GlutaMaxTM (ThermoFisher Scientific, Waltham, MA, USA), with 10% FBS (*v*/*v*) (ThermoFisher Scientific). Cells were passaged using Accutase solution (Biolegend, San Diego, CA, USA) after confluence reached 70–80%. Cells used were Mycoplasma free as assayed with PCR Mycoplasma Test (Biomedica, Piaseczno, Mazowieckie, Poland) and did not exceed 15th passage.

### 4.2. Two-Dimensional Cell Culture and Spheroid Formation

Two-dimensional cultures were maintained under standard culture conditions (5% CO_2_, 21% pO_2_). RenCa 6600 cells/cm^2^, B16F10 3300 cells/cm^2^, were seeded and cultivated for 5 days. For obtaining spheroids 500 cells were resuspended in 20 µL medium supplemented with 0.25% methylcellulose (*v*/*v* in medium), (Minneapolis, MA, USA) and seeded as a single hanging drop, on the cover of culture plate. Drops were kept in standard culture conditions (5% CO_2_, 21% pO_2_) for 72 h. After that time, the drops were individually transferred to a 96-well plate. The plates were previously covered with 1% agarose dissolved in double distilled water (*w*/*v*, VWR, Warsaw, Belgium). Cells aggregates expanded for another four-day period.

### 4.3. Western Blot

After harvesting the two-dimensional cell cultures and spheroids, the cells were PBS-washed. The cell suspensions were lysed by RIPA lysis buffer including proteases and phosphatases inhibitors (both from ThermoFisher Scientific, Rockford, IL, USA). Lysates were centrifugated to remove insoluble material (14,000× *g* for 10 min at 4 °C). Separation of equal amount of total proteins was performed using SDS polyacrylamide gels for 45 min at a constant 200 V using a Bio-Rad Minigel System. Separated proteins were transferred to nitrocellulose membranes (GE Healthcare life science, Munich, Germany). Membranes, after blocking, were incubated with primary antibodies: mTor (1:1000, Polyclonal; Cell Signaling, Warsaw, Poland), MDR1 (1:1000 anti-mo; Clone: D3H1Q, Cell Signaling, Warsaw, Poland), Vinculin (1:1000 anti-mo, clone: V284; Santa Cruz Biotechnology, CA, USA)—loading control. Bands were detected using Luminol as HRP substrate (Santa-Cruz, CA, USA) with X-ray films after treating with secondary antibodies conjugated with horseradish peroxidase-HRP (goat anti—mouse/anti-rabbit, 1:10,000; Vector Laboratories, Janki, Poland). Bands were analyzed using ImageJFiji. All data were normalized to 2DN condition.

### 4.4. Next Generation Sequencing (NGS)

For Next Generation Sequencing, total RNA was isolated using RNeasy Mini Kit (Qiagen, Hilden, Germany), according to the manufacturer’s instructions with subsequent DNA cleanup with TURBO DNA-free Kit (Thermo Fisher Scientific, Vilnius, Lithuania). Concentration of obtained RNA was measured with RNA BR Assay Kit (Thermo Fisher Scientific, Singapore), while its quality and integrity- with Qubit RNA IQ Assay Kit (Thermo Fisher Scientific, Singapore). Samples with IQ > 8.5 only were used for sequencing. cDNA libraries were obtained from mRNA, by purifying 1µg of total RNA with NEBNext^®^ Poly(A) mRNA Magnetic Isolation Module (New England Biolabs), using Ultra RNA Library Prep Kit for Illumina (Biolabs, Ipswich, MA, USA) and NEBNext Multiplex Oligos for Ilumina (Biolabs, Ipswich, MA, USA), per producers protocols. Libraries were purified with NEBNext Sample purification Beads (Biolabs, Ipswich, MA, USA) and their quality was confirmed by High Sensitivity DNA Kit (Agilent, Santa Clara, CA, USA) and Bioanalyzer (Agilent, Santa Clara, CA, USA). The sequencing was performed as outsourced service—by Lexogen GmbH (Vienna, Austria), using NextSeq 500 system (Illumina, San Diego, CA, USA). Single-end reads were aligned to Ensembl GRCm38 mouse genome with STAR (v2.7). Counts were obtained using featureCounts (v1.6.3). Differently expressed genes were identified with edgaR package (v3.3) in *R* (v4). Differentially expressed genes (DEGs) were identified by –1.5 <logFC> 1.5 and *p*-value < 0.5. Gene symbols were translated into UniProt accession numbers using the UniProt Knowledgebase (UniProtKB). Protein networks were constructed in the STRING (Search Tool for the Retrieval of Interacting Genes) database using the list of protein accession numbers as a query and then analyzed using the Cytoscape software.

### 4.5. Real Time -Polymerase Chain Reaction

RNA isolation, measuring, and purification were performed as described above. To obtain cDNA which was used in RT-PCR reaction, reverse transcription process was performed, using a commercially available High-Capacity cDNA Reverse Transcription Kit (Thermo Fisher Scientific, Vilnius, Lithuania ) according to the manufacturer’s instructions. RT-PCR reactions were performed using TaqMan Gene Expression Master Mix (Thermo Fisher Scientific, Vilnius, Lithuania ) together with commercially available probes all from Thermo Fisher Scientific: *mTor: Mm00444968_m1.*, *Actinβ: Mm02619580*. The samples run on Bio-Rad CFX384 qPCR System or CFX Connect qPCR System (Bio-Rad, Hercules, CA, USA). Amplification conditions were as follows: 50 °C (2 min), 95 °C (10 min) and 40 cycles of 95 °C (15 s) and 60 °C (1 min). The quantity of mRNA was calculated using the 2(-Delta C(T)) method and normalized to *β-actin*. All reactions were performed as triplicates. All data were normalized to 2DN condition.

### 4.6. Drug Sensitivity Assay

Anticancer drug sensitivity of B16F10 and RenCa cells dependence of microenvironment was analyzed using distinct culture conditions. Four types of drugs were tested: the mTOR inhibitor Everolimus (Lclabolatories, Woburn, MA, USA), the p-Glycoprotein inhibitor of MDR Tariquidar (APExBio, Houston, USA), the DNA intercalator Doxorubicin (Sigmaaldrich, Darmstadt, Germany), and the Cisplatin as DNA Guanine binder and replication blocker (Sigmaaldrich, Darmstadt, Germany). The cells were grown in 96-well plates according to the procedure mentioned above. At last day of cell growth, the drugs mixed with culture medium were added at final testing concentrations: Everolimus (2, 5, 10, 15, 17, 19, 25 µM), Tariquidar (10, 25, 50, 60, 80, 100, 150 µM), Doxorubicin (0.001, 0.025, 0.05, 0.1, 2 µM), and Cisplatin (1, 3, 10, 15, 20). Cell viability was monitored after 48 h of culture with the drugs using alamarBlue reduction activity of living cells (DAL1025, Invitrogen Co., Paisley, UK). A volume of 12.2 μL of alamarBlue was added. The cells cultured in 2D were incubated with the reagent for 4 h, while incubation was maintained during 12 h for cells cultured in three dimensions at 37 °C, 5% CO_2_. Reaction was measured spectrophotometrically using a Microspectrophotometer-Multiskan™ GO Microplate Spectrophotometer (570 nm and 600 nm).

### 4.7. Statistical Analysis

The results are shown as a mean +/− SEM. All data were normalized to 2DN condition. All experiments were performed at least in 3 biological replicates. All statistical analyses were performed using the GraphPad Prism 9.0 software (RRID:SCR_002798). Depending on the Gaussian distribution, we performed One-Way Analysis of variance (ANOVA) with Tukey post-hoc test or Kruskal–Wallis with post-hoc Dunn’s test.

## 5. Conclusions

We here observed a distinct susceptibility to doxorubicin or everolimus and cisplatin in spheroid models of two cancer types. They differed in the expression of MDR1 and Cyp2s1, but other drug resistance mechanisms were similarly induced in both models. We excluded the limited drug penetration related to 3D architecture as a cause of lower cell responsiveness to drugs. Other features linked to cancer type/cell line intrinsic characteristics might be responsible for the outcome of the MDR phenotype. Although we would not identify the molecular changes that could predict drug resistance, our model serves as an interesting tool for experimental testing of anti-cancer candidates. Such 3D culture can be potentially used for in vitro testing using patient-derived cells, as a clinically relevant and easily accessible instrument for personalized medicine, in the face of lack of molecular prediction markers.

## Data Availability

All data generated or analyzed during this study are included either in this article or in the Supplementary Figures. The data that support the findings of this study are available from the corresponding author upon reasonable request. NGS data have been deposited in NCBI’s Gene Expression Omnibus [60] and are accessible through GEO Series accession number GSE190296 (https://www.ncbi.nlm.nih.gov/geo/query/acc.cgi?acc=GSE190296) (accessed on: 6 December 2021).

## Supplementary Materials

The following are available online at https://www.mdpi.com/article/10.3390/ijms23031166/s1.
