# Supplementary material for: Spheroid Culture Differentially Affects Cancer Cell Sensitivity to Drugs in Melanoma and RCC Models
Source: Int J Mol Sci. 2022 Jan 21;23(3):1166. doi: 10.3390/ijms23031166 (PMC8835769; doi:10.3390/ijms23031166)
Supplement: Supplementary file 1 [file ijms-23-01166-s001.zip › ijms-1523149-supplementary.pdf]

Supplementary Material

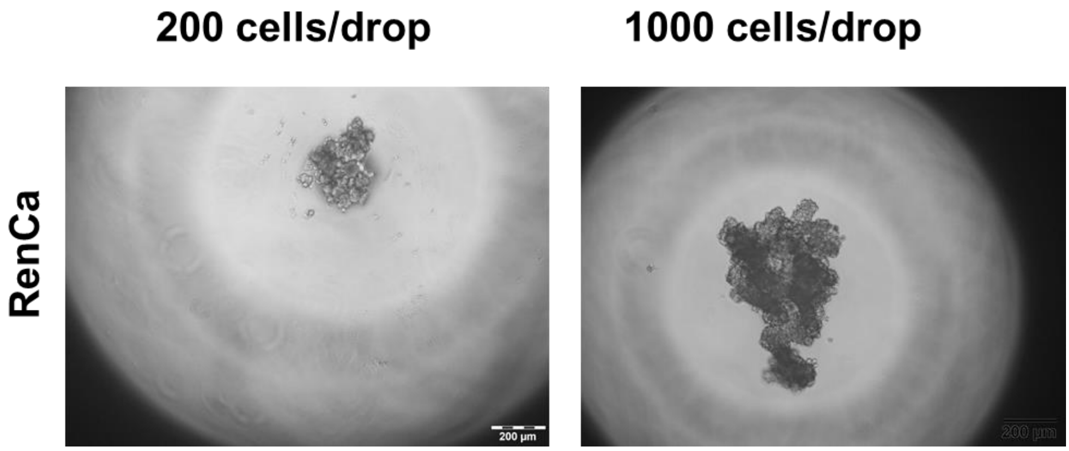

Supplementary Figure S1. **Optimalization of spheroid cultures of RenCa cells.** Cell aggregates after 3 days of cultures in hanging drops in different seeding densities.

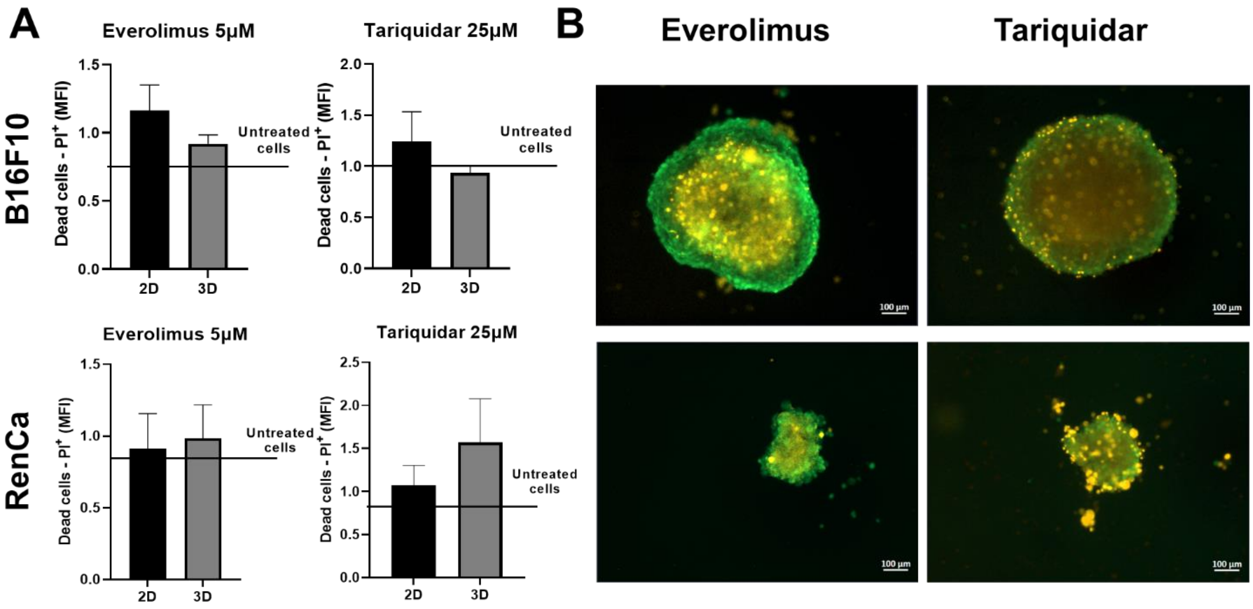

Supplementary Figure S2. **Cell sensitivity in 2D and 3D culture conditions to everolimus and tariquidar.** A—Sensitivity to everolimus and tariquidar of B16F10 and RenCa cells in 2D and 3D models assessed by quantification of propidium iodide incorporation by fluorescence microscopy. B—Fluorescence staining of spheroids by propidium iodide (orange, dead cells) and calcein (green - live cells). Statistical analysis was performed by One-Way ANOVA/Tukey test—*p<0,05, N=3 (2D-control).

Drug sensitivity assay—Methodology

AlamarBlue assay results for viability were further compared to fluorescence microscopy assessment data. The cells were grown in 96-well plates according to the procedure mentioned in the text. On the last day of cell growth, the drugs mixed with culture medium were added to reach final testing concentrations of: Everolimus 5µM, Tariquidar 25µM. After 48h, microscopy imaging was performed using calcein acetoxymethyl ester (Calcein AM Biolegend) which is hydrolyzed by cellular esterases to free fluorescent calcein in the cytoplasm and propidium iodide DNA intercalating agent, highly fluorescent after intercalation between the DNA bases (Exbio, Czech Republic). Briefly, after adding the dyes, spheroids and cells grown in 2D were incubated for 15 minutes in the dark, at room temperature. Images were acquired using a Zeiss AxioObserver.7, fluorescence, and inverted microscope (5X magnification) and analysis performed with the Zen 2.6 blue edition software (Zeiss, Germany). The sensitivity to drugs was assessed by comparing the propidium iodide fluorescence intensity of treated cells to untreated control (ImageJ Fiji).
